# Dynamic Roles of Insect Carboxyl/Cholinesterases in Chemical Adaptation

**DOI:** 10.3390/insects14020194

**Published:** 2023-02-16

**Authors:** Casey Cruse, Timothy Walter Moural, Fang Zhu

**Affiliations:** 1 Department of Entomology, Pennsylvania State University, University Park, State College, PA 16802, USA; 2 Huck Institutes of the Life Sciences, Pennsylvania State University, University Park, State College, PA 16802, USA

**Keywords:** metabolic detoxification, hydrolysis, sequestration, catalytic triad, pesticide resistance, olfaction, odorant-degrading enzymes

## Abstract

**Simple Summary:**

Carboxyl/cholinesterases (CCEs) represent a family of enzymes distributed in many organisms, including insects. Despite their relatively simple catalyzed hydrolysis reaction, CCEs facilitate insects’ adaptation to chemical signals and stressors from the environment through various trajectories, including developing pesticide resistance, facilitating the adaptation of insects to their host plants, and manipulating insect behaviors. The CCE-mediated mechanisms of pesticide resistance to organophosphate, carbamate, or pyrethroid pesticides comprise enhanced metabolism, the sequestration of pesticides to prevent them from reaching their target sites, or conformational changes in target sites to prevent pesticides from binding. In addition, CCEs aid in the adaptation to chemical signals through the olfactory system by degrading insect semiochemicals. In this review, we summarize the current knowledge of the classification, structures, and functions of insect CCEs, which will help the development of more sustainable pest control strategies in the future.

**Abstract:**

Insects have evolved several intricate defense mechanisms to adapt to their chemical environment. Due to their versatile capabilities in hydrolytic biotransformation, insect carboxyl/cholinesterases (CCEs) play vital roles in the development of pesticide resistance, facilitating the adaptation of insects to their host plants, and manipulating insect behaviors through the olfaction system. CCEs confer insecticide resistance through the mechanisms of qualitative or quantitative changes of CCE-mediated enhanced metabolism or target-site insensitivity, and may contribute to the host plant adaptation. CCEs represent the first odorant-degrading enzymes (ODEs) discovered to degrade insect pheromones and plant odors and remain the most promising ODE candidates. Here, we summarize insect CCE classification, currently characterized insect CCE protein structure characteristics, and the dynamic roles of insect CCEs in chemical adaptation.

## 1. Introduction

Pesticides are the mainstay for the control of numerous pest populations in agricultural and urban ecosystems. However, the extensive application of pesticides accelerates the accumulation of resistance-associated genes in survivors and causes the development of pesticide resistance. Commonly, multiple mechanisms are involved in pesticide resistance, including changes in cuticle thickness and composition, behavioral avoidance, target site insensitivity, sequestration, and metabolic detoxification [1]. With the advance of genomic and post-genomic technologies, studies uncovering the genes, pathways, mechanisms, and ecological factors responsible for the evolution of pesticide resistance become promising. Further research on pesticide adaptation is of theoretical and applied importance in understanding the evolution of resistance and in helping the development of more sustainable pest control strategies in the future.

Insects have evolved sophisticated mechanisms to adapt to various xenobiotics, such as plant allelochemicals, insect odors and pheromones, pesticides, and industrial pollutants [1,2,3]. Metabolic detoxification is a multi-phase process involving the enzymatic degradation and conjugation of lipophilic compounds to water-soluble, excretable metabolites [3,4,5,6]. In Phase I, cytochrome P450 monooxygenases (P450s) and carboxyl/cholinesterases (CCEs) convert xenobiotics from more lipophilic compounds to more hydrophilic products. In Phase II, glutathione S-transferases (GSTs) and/or UDP-glycotransferases (UGTs) further conjugate the xenobiotic metabolites or Phase I products and prepare them for excretion. In Phase III, the products can be excreted through cellular transporters, such as ATP-binding cassette (ABC) transporters [5,7,8].

CCEs constitute a multigene family of α/β-hydrolase fold enzymes that are distributed in numerous organisms including insects, mammals, plants, and microorganisms [9,10,11,12,13]. As Phase I enzymes, insect CCEs hydrolyze structurally diverse xenobiotics containing ester or amide bonds that consist of pesticides, insect and plant odors, insect pheromones and hormones, as well as environmental toxicants [9,14,15,16]. Due to their versatile capabilities in hydrolytic biotransformation, insect CCEs play vital roles in the development of pesticide resistance, facilitating the adaptation of insects to their host plants and manipulating insect behaviors through olfaction [9,17,18]. In this review, we summarized the current knowledge of insect CCE classification, protein structure characteristics, and the dynamic roles of insect CCEs in chemical adaptation.

## 2. CCE Classification and Structural Characteristics

### 2.1. Classification of Insect CCEs

Insect CCEs are divided into 14 clades in three classes based on their physiological and biochemical functions: dietary/xenobiotic detoxification (clades A–C), pheromone/hormone processing (clades D–H), and neuro/developmental functions (clades I–N) (Table 1) [9,19,20]. The dietary detoxification class of CCEs is of the highest quantity and sequence variation among species. CCEs in this class are identified as intracellular catalytically active enzymes and belong to α-esterases (Table 1) [19,20]. There are three major clades in this class, the A–C clades. Most insect species have the clade B CCEs (microsomal α-esterases) in their genomes [20].

There are five major clades in the secreted, catalytically active pheromone/hormone processing class: integument esterases (Clade D), β-esterases (Clade E), juvenile hormone esterases (JHEs) (Clades F and G), as well as glutactin and similar enzymes (Clade H) (Table 1) [19,20]. Clade D is composed of one to several integument esterases or semiochemical esterases in each insect genome (Table 1). The functions of integument esterases are implicated in pheromone and other semiochemically triggered signaling processes [15,19,21,22,23,24]. Recent studies have suggested that some insect integument esterases may contribute to xenobiotic detoxification [14,25]. β-esterases in Clade E exhibit a wide range of functions that have been extensively studied [19,20]. For example, the E4 and FE4 esterases in *Myzus persicae* confer organophosphate (OP) resistance to the resistant populations [26,27]. *Apol*PDE, *Pjap*PDE, and Est-6, male specific β-esterases in *Antheraea polyphemus*, *Popillia japonica*, and *Drosophila melanogaster*, respectively, are involved in the termination of sex pheromones [28,29,30]. Some β-esterases, such as SexiCXE14 from *Spodoptera exigua*, are reported to degrade both plant volatiles and sex pheromones [31]. One honey bee β-esterase, GB15327, was proposed to be a JHE because the functional studies indicate that this β-esterase affects JH metabolism [32]. JHEs (Clades F and G) are typically classified into two distinct clades in phylogeny—lepidopteran type JHEs and dipteran type JHEs [20]. In general, lepidopteran type and dipteran type JHEs share the GQSAG and GxxHxxD/E motifs. The serine and histidine residues in these two motifs form part of the catalytic triad that is critical for JH-specific esterase activities [33,34]. JHEs are a few of the insect CCEs that have been relatively well characterized by function, due to their specific function on the clearance of JH, which is the critical sesquiterpenoid hormone in regulating insect metamorphosis and development [19,34,35,36,37,38,39].

The neuro/developmental class include the acetylcholinesterase (AChE) and five noncatalytic clades (Clades I-N) [20,40]. Aside from AChEs, many of the neuro/developmental class are membrane-bound and fall under the subclassification CLAMs (cholinesterase-like adhesion molecules), of which one or more residues of the catalytic triad are absent [41]. CLAMs include neuroligins, glioactins, neurotactins and glutactins, with the latter two only found in insects [41,42] (Table 1). The major function of AChEs in insects is the hydrolysis of the neurotransmitter, acetylcholine (ACh), after it binds to the Ach receptor (AChR) at the cholinergic synapses [19]. Mutations on AChE can inhibit OP and carbamate toxicities and have been linked with OP and carbamate resistance in many insect species [43,44,45,46,47]. Except for AChEs, there are few detailed studies on other genes in the neuro/developmental class in insects, mainly in *D. melanogaster* [19,42,48,49].

### 2.2. Structural Characteristics of Insect CCEs

All currently characterized insect CCEs contain the canonical α/β hydrolase fold backbone structure, with six interspersed α-folds distributed about a core β-sheet that contains eight β-strands, with seven of the core β-strands running in the parallel direction and one β-strand in anti-parallel formation [50]. Additional β-strands, helices, and coils surround the core α/β hydrolase fold but vary across clades. This conserved backbone maintains the position of the enzyme’s active site: the catalytic triad and the oxyanion hole. In turn, the catalytic triad is the essential component in the CCE hydrolysis reaction [9,13]. The six insect CCE crystal structures resolved to date have a catalytic triad consisting of a serine, a glutamate and a histidine, except for Est6 from *D. melanogaster* with an aspartate in place of the glutamate (Table 2) [37,51,52,53,54]. During the catalyzed ester hydrolysis reaction, the ester bond of the substrate is cleaved, resulting in the formation of an alcohol and a carboxylic acid product. To start the reaction, the acidic residue of the catalytic triad (Glu or Asp) is hydrogen bonded to the His, stabilizing it as a general base and allowing it to deprotonate the nucleophilic Serine. The nucleophilic Serine then attacks the carbonyl carbon of the bound ester substrate, forming a tetrahedral intermediate that is, in turn, stabilized by the oxyanion hole. Next, the tetrahedral intermediate collapses, deprotonating the Histidine and releasing the alcohol product, and forming the acyl-enzyme intermediate. The Histidine then deprotonates a water molecule, activating it to attack the carbonyl carbon of the acyl-enzyme intermediate, releasing the acid product and returning the enzyme to its resting state. Often, the structure of the CCE active site allows for the metabolism of many substrates, but preferences in CCEs for specific xenobiotics are observed [9,13,37].

**Figure 1:**
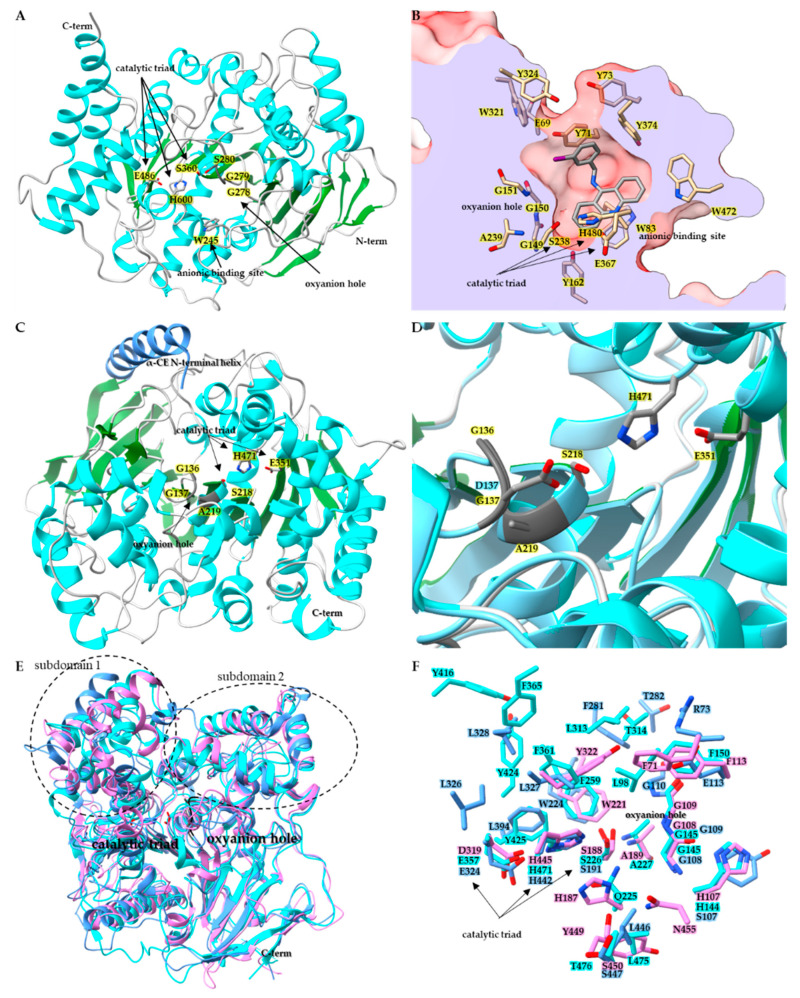
Structures of insect CCEs. (**A**) Ribbon diagram of acetylcholinesterase (PDB: 6ARX) from *Anopheles gambiae*. Side chains for key active site residues are shown and colored by element: catalytic triad Ser360, Glu486, His600, and oxyanion hole residues Gly278, Gly279, Gly280Ser mutation, and the anionic binding site Trp245. (**B**) Surface representation of acetylcholine binding pocket of *Dm*AChE PDB:1QON, clipped to show active site gorge. Active site gorge residues are colored by heteroatom and the bound inhibitor 9-(3-ioodobenzylamino)-1,2,3,4-tetrahdroacridine is colored by element. (**C**) Ribbon diagram of wild-type *Lc*αE7 PDB:4FNG, showing catalytic triad side chains colored by element, and oxyanion hole colored dark gray. (**D**) Active site of *Lc*αE7, wild-type structure PDB:4FNG is superimposed with mutant Gly137Asp PDB:5C8V. (**E**) Superimposed ribbon diagrams of juvenile hormone esterase *Ms*JHE PDB:2FJ0 (colored in cyan), with β-esterases *Cq*ESTβ2 PDB:5W1U (colored in cornflower blue) and *Dm*EST6 PDB:5THM (colored in orchid). Catalytic triad residues sidechains are shown and the oxyanion hole portion of the ribbon diagram is colored black. (**F**) Zoomed in binding pocket residues for the superimposed *Ms*JHE PDB:2FJ0, *Cq*ESTβ2 PDB:5W1U and *Dm*EST6 PDB:5THM structures with residues colored by heteroatom.

Belonging to the neuro/developmental class, insect AChE (E.C. 3.1.1.7) contains the core α/β hydrolase that is conserved across species (Figure 1A) [55]. A comparison of superimposed AChE catalytic domains from *Anopheles gambiae* (*Ag*AChE PDB:6ARX), *D. melanogaster* (*Dm*AChE:PDB 1QO9), *Homo sapiens* (*h*AChE:PDB 4EY4), *Mus musculus* (*m*AChE PDB:2HA2) and *Torpedo californica* (*Tc*AChE PDB:2WG2) revealed α-carbon backbone root-mean-square-deviation (RMSD) ranges from 1.1 Å to 1.9 Å [55,56]. A key structural characteristic of AChE is the active site gorge, a channel starting at the surface of the AChE and extending deep into the protein and terminating at the catalytic/acetylcholine binding site (Figure 1B) [52]. In the *Dm*AChE:PDB 1QON crystal structure with inhibitor (1,2,3,4-tetrahydro-N-(3-iodophenyl-methyl)-9-acridinamine) bound in the acetylcholine binding site, there is a 17.7 Å distance from the hydroxyl of Tyr73 at the entrance of the active site gorge to the hydroxyl of Tyr162 at the bottom of the gorge, below the catalytic triad. For *Ag*AChE PDB:6ARY, the distance from the γ^2^ carbon of Val235 (positionally equivalent to Tyr73 in *Dm*AChE) at the gorge entrance to the hydroxyl of Tyr291 (equivalent to Tyr162 in *Dm*AChE) is 17.9 Å [52,56]. Much of the AChE active site gorge is lined with aromatic residues thought to aid the delivery of the substrate to the catalytic site containing the catalytic triad, oxyanion hole and the conserved anionic binding site tryptophan [56]. In *Dm*AChE, the catalytic triad is made up of Ser238, His480, and Glu367; adjacent to the triad is the oxyanion hole composed of Gly150, Gly151, and Ala239 backbone amides, and the conserved anionic binding site Trp83 (Figure 1B) [52]. Multiple instances of a G280S mutation in *Ag*AChE located in the oxyanion hole have been documented (Figure 1A) [56]. Structural analysis by Cheung et al. revealed that the G280S mutation in the oxyanion hole results in steric crowding of the acetylcholine binding site, with the hydroxyl of S280 pointing towards the catalytic triad, and as a result steric crowding leads to the loss of target site sensitivity towards large OP and carbamate insecticides [56].

**Table 1:** The information of CCE genes in 24 insect species.

Order	Species	Dietary/ Detoxification	Hormone/Semiochemical Process				Neurodevelopmental Process						Total	Reference
		α-Esterase and Others (Clades A–C)	Integument Esterase (Clade D)	β-Esterase (Clade E)	JHE (Clades F and G)	Glutactin and Like Enzymes (Clade H)	Uncharacterized (Clade I)	AChE (Clade J)	Uncharacterized Neuroreceptors (Clade K)	Neuroligin (Clade L)	Gliotactin (Clade M)	Neurotactin (Clade N)		
Coleoptera	*Leptinotarsa decemlineata*	52	8	3	1	1	1	2	1	2	1	0	72	[25]
	*Tribolium castenaum*	26	3	4	2	1	1	2	2	5	1	0	47	[25]
Diptera	*Aedes aegypti*	22	1	2	12	10	0	2	0	0	0	0	49	[57]
	*Anopheles gambiae*	16	0	4	10	10	1	2	1	5	2	0	51	[20]
	*Culex quinquefasciatus*	30	1	3	22	6	0	2	1	3	1	2	61	[40]
	*Drosophila melanogaster*	13	3	2	3	5	1	1	1	4	2	0	35	[20,58]
	*Musca domestica*	17	7	2	1	5	0	1	1	3	2	0	39	[14]
	*Sphaerophoria rueppellii*	15	0	9	4	4	0	1	0	5	1	1	40	[59]
Hemiptera	*Bemisia tabaci*	6	0	15	3	1	1	4	1	10	1	0	42	[58]
	*Diaphorina citri*	2	0	2	4	3	0	2	1	6	0	0	20	[60]
	*Myzus persicae*	5	0	12	0	0	1	3	1	0	0	0	22	[61]
	*Nilaparvata lugens*	3	1	19	0	2	0	2	0	1	1	0	29	[62,63]
	*Orius laevigatus*	0	0	6	9	1	1	2	1	8	3	1	32	[64]
	*Pediculus humanus*	3	0	1	0	1	0	2	1	6	3	0	17	[65,66]
	*Rhodnius prolixus*	0	0	40	0	2	1	2	1	13	2	0	61	[67]
	*Triatoma dimidiata* *	0	0	25	0	1	0	0	0	1	0	0	27	
	*Triatoma infestans* *	0	0	18	0	0	0	1	0	0	0	0	19	
	*Triatoma pallidipennis* *	0	0	17	0	1	0	0	0	0	0	0	18	
Homoptera	*Acyrthosiphon pisum*	5	0	18	0	0	1	2	1	3	0	0	30	[61]
Hymenoptera	*Apis mellifera*	8	1	3	1	0	2	2	1	5	1	0	24	[68]
	*Nasonia vitripennis*	13	4	11	2	1	1	2	1	5	1	0	41	[20]
Lepidoptera	*Bombyx mori*	55	0	2	4	0	1	2	1	6	1	2	74	[69]
	*Plutella xylostella*	20	1	1	6	1	4	2	6	4	3	0	48	[70,71]
	*Spodoptera litura*	83	4	2	8	0	0	2	3	7	1	1	111	[72]

* Data are from normalized transcriptomes and the CCE numbers may be underrepresented.

**Table 2:** The information of CCE genes in insect species that have 3D structures available.

Class	Clade	Species Name	Protein Name	Catalytic Triad	Reference
Dietary/xenobiotic detoxification	α-esterases	*Lucilia cuprina*	*Lc*αE7 (PDB: 4FNG)	Serine, glutamate, and histidine	[51]
Hormone/pheromone processing	β-esterases	*Drosophila melanogaster*	*Dm*Esterase-6 (PDB: 5THM)	Serine, aspartate, and histidine	[73]
		*Culex qunquefasciatus*	*Cq*estβ2 (PDB: 5W1U)	Serine, glutamate, and histidine	[53]
	JHEs	*Manduca sexta*	*Ms*JHE (PDB: 2FJ0)	Serine, glutamate, and histidine	[37]
Neurodevelopmental	AChEs	*Drosophila melanogaster*	*Dm*AChE (PDB: 1QO9)	Serine, glutamate, and histidine	[52]
		*Anopheles gambiae*	*Ag*AChE (PDB: 5X61)	Serine, glutamate, and histidine	[55,56]

Insect CCEs are known to play multiple physiological roles, such as hormone, neurotransmitter, pheromone, odorant, lipid ester, and insecticide degradation [51,73,74,75]. Multiple instances of insecticide resistance have been reported resulting from overexpression and/or mutation events in α-esterases [51,74]. The inactivation of esterase enzymes can occur upon exposure to OPs via phosphorylation of the active site serine [76]. Mutations that alter the active site can lead to the gained ability of the enzyme to overcome inactivation upon exposure to OPs. A notable mutation has been found to occur in an α-esterase belonging to the dietary/xenobiotic detoxification class (Figure 1C,D) [74]. The G137D mutation found in sheep blowfly *Lucilia cuprina* α-esterase *Lc*αE7 caused gaining of function hydrolysis activity toward the OP chlorfenvinphos, and in turn resulted in lost activity towards model substrates α-naphthyl acetate, p-nitrophenyl acetate, and methylthiobutyrate (Figure 1D) [74]. In a follow up study by Jackson et al. [51], protein crystallography was used to determine the structure of *Lc*αE7, results of which suggested the probable original wild-type *Lc*αE7 natural substrates to be fatty acid methyl esters (Figure 1C) [51]. As with AChE, β-esterase and JHE, the α-esterase *Lc*αE7 contained the common esterase structural motifs. However, in addition to the typical α/β core structure, a new helix on the n-terminus was observed that could serve as a membrane anchor (Figure 1C). *Lc*αE7 possessed a catalytic triad, consisting of Ser218, His471, and Glu351, along with an oxyanion hole composed of Ala219, Gly136, and Gly137. The wildtype structure further suggested that the G137D mutation of *Lc*αE7 found in blowfly populations should be located in the wild-type oxyanion hole adjacent to the catalytic triad and had been shown to increases OP hydrolysis [51,74,77]. A later study revealed the crystal structure of the mutant G137D *Lc*αE7 and confirmed the location of Asp137 to be adjacent to the nucleophilic Ser218 of the triad (Figure 1D) [77]. The highly purified G137D *Lc*αE7 showed a five-fold decrease in turnover for model substrate 4-nitrophenyl butyrate and a 14-fold increase in turnover for the OP diethylumbelliferyl phosphate compared to wildtype *Lc*αE7. Newcomb et al. [74] and Mabbitt et al. [77] postulated that Asp137 acts as a general base, deprotonating a water molecule activating it for nucleophilic attack on the phosphorylated triad Ser218, and in turn the tetrahedral intermediate is protonated by His471, the tetrahedral intermediate collapses and the product is released, completing the hydrolysis of the OP and returning *Lc*αE7 to the resting state [77]. The case of the G137D *Lc*αE7 is a good example of insecticide usage leading to a change in population genetics. The G137D mutation resulted in a structural change of the catalytic site and the gain of function towards a new environmental stress; in this case, man-made OPs. It has been reported that as many as 95% of the individuals in some *L. cuprina* populations now possess the G137D *Lc*αE7 mutation [77,78,79].

The hormone/pheromone processing class contains the insect β-esterase and juvenile hormone esterase [37,53,73]. Crystal structures for *Cq*ESTβ2 (PDB:5W1U), *Dm*EST6 (PDB:5THM), and *Ms*JHE (PDB:2FJ0) revealed that the hormone/pheromone processing class exhibits the typical esterase structural elements (Figure 1E and 1F). When the structures for *Cq*ESTβ2 (PDB:5W1U), *Dm*EST6 (PDB:5THM) and *Ms*JHE (PDB:2FJ0) are superposed with *Dm*AChE PDB:1QON, the largest α-carbon RMSDs are observed in N and C terminal regions, along with the subdomain 1 and subdomain 2 regions adjacent to the catalytic triad that play a dominant role in defining the size and shape of the binding pocket and thus the substrate specificity of CCEs [37,52,53,73]. When the insect CCE binding pockets were compared and examined, *Ag*AChE was found to have a binding pocket volume of 1736.2 Å, *Ms*JHE pocket volume was 1308.0 Å, *Lc*αE7 pocket volume was 2725.5 Å, *Dm*EST6 pocket volume of 935.0 Å, and *Cq*ESTβ2 pocket volume was 4735.5 Å [53]. The observed size and shape of the insect CCE are complementary to their preferred substrates: *Ag*AChE and acetylcholine, *Ms*JHE and juvenile hormone, *Lc*αE7 and fatty acid methyl esters, *Dm*EST6 and short to medium chain length food odorant esters, and *Cq*ESTβ2 with mono or diacylglycerols of medium chain length [53]. In the case of *Cq*ESTβ2, it was found to play an insecticide resistance role in *Culex quinquefasciatus* through a sequestration mechanism, exhibiting a high affinity for OPs but also a low turnover number [53].

## 3. Dynamic Rules of Insect CCEs in Chemical Adaptation

The function and specificity of individual CCEs can be defined by their substrate binding affinity and the rate at which they are able to catalyze the hydrolysis of different esters. One of the most substrate specific is the JHE in insects [36]. Although it has a relatively slow hydrolysis speed, JHE has a high affinity for JH. The substrate specificity of JHE allows it to regulate the hemolymph titer of JH even at incredibly low concentrations [36]. Insect AChEs vary greatly in their rate of hydrolysis but are generally quite active at low substrate concentrations and inhibited by high substrate concentrations. They have only a few substrates: acetylcholine/thiocholine, propionylcholine/thiocholine and butyrylcholine/thiocholine [80]. Odorant-degrading enzyme CCEs, especially those specific to pheromones, often have a high binding affinity and hydrolysis rate for their preferred substrates, leading to rapid odorant degradation even in low substrate concentrations. This rapid hydrolysis is critical in preventing odor molecules from continually binding to antennal receptor neurons and creating a false detection of odors that are no longer in the air space of the insect [28,81].

Deciphering the biochemical functions of insect CCEs and their substrate spectrums is vital for understanding the mechanisms by which insects adapt to their chemical environment. Gains in our understanding of such mechanisms will the facilitate management of pesticide resistance in insect pests and aid in protecting pollinators and other beneficial insects from xenobiotics. The current section will synopsize mechanisms of xenobiotic adaptation associated with insect CCEs that are involved in metabolizing pesticides, plant allelochemicals, and degrading volatile semiochemicals.

### 3.1. CCE-Mediated Insecticide Resistance

Insect CCE, cytochrome P450 and GST enzyme families are commonly implicated in the development of insecticide resistance [82]. Most enhanced CCE mediated detoxification is detected in OP and/or carbamate resistant populations. In some cases, enhanced CCE detoxification is also involved in the resistance to pyrethroids, by hydrolyzing or sequestering insecticides before they reach their target: voltage-gated sodium channels [83]. The mechanisms of CCE-mediated resistance to these insecticides include quantitative changes (enhanced CCE gene expression and enhanced CCE activities) and qualitative changes (mutations occur in the active sites) [17,84,85]. OPs and carbamates are degraded through a hydrolysis reaction similar to CCE natural substrate hydrolysis. Qualitative changes in CCEs can enhance OP metabolism through a mutation that increases OP hydrolysis ability, and in turn reduces a CCE’s natural substrate hydrolysis activity. Quantitative changes arise through an enhanced expression of a CCE that can bind an insecticide, but with little or no hydrolysis activity (i.e., sequestration) [53,85]. The enhanced CCE expression can occur through mutations in the regulatory sequence (*cis*- or *trans*-), or gene amplification. The enhanced CCE expression (quantitative changes) can also be combined with mutations in the coding sequence (qualitative changes) that cause boosted metabolism of insecticides or increased binding affinity, resulting in higher levels of insecticide resistance [17]. Insensitivity of AChE is shown to arise through one or more mutations in the *Ace* genes, which encode insect AChEs. Multiple mutations often lead to greater insensitivity and resistance than one mutation [86,87]. The following examples demonstrate these CCE-associated metabolic detoxifications involved in OP and carbamate resistance and, more rarely, pyrethroid resistance.

Peach-potato aphid (*Myzus persicae*) variants showed cross resistance to OPs, carbamates and pyrethroids by upregulating the expression of β-esterases E4 or FE4 (different variants boost copies of E4 or FE4). Resistant variants had as many as 80 more copies of the gene at the genomic DNA level than the susceptible variant, with no change in the enzyme coding sequence [88,89]. E4 could hydrolyze dimethyl and diethyl OPs at a slow rate, resulting in hydrolysis of a small fraction of OPs. While the major role of E4 involved in OP resistance was through sequestration. E4 hydrolyzed carbamates at even slower rates, which was consistent with the relative resistance level that *M. persicae* had for both insecticide classes. Interestingly, E4 was also able to hydrolyze the *trans*-isomers of the pyrethroid insecticide permethrin, but not *cis*-isomers, and at a significantly lower rate than OPs and carbamates [89]. Subsequently, greater resistance to all three classes of insecticides had been detected in *M. persicae* populations through both E4 enhanced expression and target-site insensitivity (a mutation in the AChE enzyme or the voltage gated sodium channels) [90,91]. A similar mechanism of resistance was identified in the *Nl*-EST1 gene in the brown planthopper, *Nilaparvata lugens*, which shares 38% sequence identity (higher than other insect CCEs) with *M. persicae* E4. Similar to E4, the *Nl*-EST1 gene expression was enhanced through gene amplification in OP and carbamate resistant variants, while it showed no change in the enzyme coding sequence between the resistant and susceptible variants. The gene copies from amplification were less than that of E4 and correspond with the relative resistance level [92,93].

In a diazinon OP resistant variant of the sheep blowfly *L. cuprina*, one mutation (Gly to Asp) within the active site of an α-esterase *Lc*αE7 (E3) was detected to enhance the hydrolysis of diethyl OPs and subsequently reduce the CCE natural substrate hydrolysis activity [74]. The same amino acid substitution Gly to Asp was associated with diethyl OP hydrolysis and reduced CCE hydrolysis activity in the house fly *Musca domestica* [74,94]. An alternative mutation in an allele of E3 was detected in a malathion OP resistant population of *L. cuprina*, with a change from Trp to Leu close to the catalytic triad. This mutation allows for greater malathion hydrolysis in malathion resistant *L. cuprina* populations than susceptible ones, which show malathion hydrolysis but at very low levels. When the mutant E3 was phosphorylated by the oxidized malathion, malaoxon, it could reactivate and continue hydrolyzing malathion and malaoxon, whereas susceptible variant malathion hydrolyzing CCEs were irreversibly inactivated by phosphorylation [95].

The fungus gnat *Bradysia odoriphaga*, which in its larval form is a crop pest of the Chinese chive, has been known to develop insecticide resistance in field populations. The CCE *Bo*αE1 of the dietary/detoxification class, isolated from a laboratory colony, was transcriptionally upregulated after malathion exposure and showed hydrolysis capability for malathion. The induced upregulation of *Bo*αE1 was suggested to contribute to the resistance found in field populations [96]. In *C. quinquefasciatus*, a CCE *Cq*estβ2 was upregulated and played a major role in OP resistance through acting as a “sponge” to intercept insecticides before reaching their target. The stopped-flow kinetic analysis showed that a covalent intermediate formed after the rapid OP binding of *Cq*estβ2 with high affinity, resulting in irreversible and prolonged enzyme inhibition [53]. By contrast, a duplication and series of mutations in a malathion resistant *Culex tarsalis* generated a new malathion carboxylesterase (MCE I) that could hydrolyze malathion 18 times faster than MCE II. MCE II was in both the susceptible and resistant strains and neither MCE I nor MCE II showed higher expression, indicating that the insecticide resistance was due to qualitative changes but not quantitative changes [97]. CCE mediated insecticide resistance had also been implicated in house fly *M. domestica* through the constitutive and permethrin induced upregulation of multiple α-esterases, β-esterases, and integument esterases [14,98], and in tropical bedbug *Cimex hemipterus* through OP and carbamate induced upregulation [99], with a significant increase in overall esterase activity between populations sampled in 2002 and 2016 [100]. Recently, one CCE04^SY-VP^ was functionally characterized in two genetically independent spirodiclofen resistant *Tetranychus urticae* strains. Studies suggested that the sequestration of spirodiclofen by overexpressed CCE04^SY-VP^ was likely to contribute to the spirodiclofen resistance in these two strains [101]. More recently, the constitutive and inductive overexpressions of several α-esterases were selected by using forward and reverse genetic approaches in multi-insecticide resistant *Plutella xylostella* strains [102]. Further functional studies suggested that the overexpression of one of these esterases, *Px*αE14, may play roles in resistance to multiple OP and pyrethroid insecticides in *P. xylostella* [103].

AChE insensitivity has developed in many insect species, including *D. melanogaster* [86], *Aphis gossypii* [104], *Leptinotarsa decemlineata* [43], *M. domestica* [105], and *T. urticae* [106]. Certain point mutations are recorded to have a desensitization effect toward a specific insecticide but have no effect towards other insecticides, or even increase sensitivity to other insecticides. Additionally, a combination of multiple mutations in AChE can result in a more complete resistance/desensitization to multiple insecticides [45]. One of the documented mutations that conferred AChE insensitivity was a single point mutation changing a Gly to a Ser in the *ace-2* gene that had independently occurred four times in different mosquito species: *C. pipiens*, *A. albimanus*, and *A. gambiae* [87].

### 3.2. Metabolism of Plant Allelochemicals

CCEs combined with GSTs and Cytochrome P450s are three major superfamilies of detoxification genes involved in plant allelochemical detoxification which facilitate insect host plant adaptation [2,107,108,109,110,111,112]. Cytochrome P450s are well known to play important roles in plant allelochemical metabolism and have been broadly studied [17,113,114]. However, the involvement of specific CCE genes in the metabolism of plant allelochemicals is rare and requires further investigation. Most studies focus on a comparison of total esterase levels in response to different plant diets, and in some cases how this affects susceptibility to insecticides.

The western tiger swallowtail *Papilio rutulus* larvae were tolerant of diets containing phenolic glycosides tremulacin and salicortin, both of which were found in one of their host plants, quaking aspen [115]. When fed phenolic glycosides in conjunction with an esterase inhibitor, S,S,S-tributylphosphorotrithioate (DEF), the growth and survival of larvae were reduced by half compared to larvae fed phenolic glycosides alone and significantly reduced compared to larvae fed DEF alone, suggesting the involvement of CCEs in the metabolism of phenolic glycosides [115]. In another species, Spongy moth (*Lymantria dispar*), feeding on a diet with added phenolic glycosides (2 or 4% wet weight) induced both CCE and GST activity in the midgut of the larvae, indicating CCEs facilitating the host plant adaptation of Spongy moth [116,117]. In *Sitobion avenae*, CCE activity was induced by indole alkaloid gramine. The increase in CCE activity was positively correlated with the concentration of gramine, suggesting that CCE may contribute to the host allelochemical adaptation [118].

In tobacco budworm, *Helicoverpa assulta*, and tobacco cutworm, *Spodoptera litura*, differences in diets and plant allelochemicals affected both the expression of detoxifying enzymes and susceptibility to insecticides [109,119,120,121]. *S. litura* showed a 4.9-fold increase in CCE activity and a 2.4-fold increase in GST activity in addition to a greater resistance to Phoxim, Methomyl, Chlorfenapyr, Fenvalerate, and Emamectin benzoate when fed with tobacco plants compared with sweet potato plants [120]. In *T. urticae*, an increase in OP dimethoate pesticide mortality was detected after feeding for seven days on cucumber versus lima bean plants. This enhanced mortality was correlated with a 60% decrease in overall esterase activity, indicating the potential roles of CCE-mediated detoxication in host plant adaptation and insecticide resistance [107].

### 3.3. Odorant Degradation in the Olfactory System

Olfaction plays vital roles in insect communication. One of the major olfactory organs in insects is the antenna, which detects volatile semiochemicals (e.g., plant volatiles, pheromones, insect odors) from the external environment for host plant location, mating, and avoiding predators [15,122]. After volatile semiochemicals bind to odorant receptors, triggering signal transduction, insect olfactory pathways have evolved to include a dynamic process of signal inactivation by odorant-degrading enzymes (ODEs) [18]. Highly effective ODEs are required for degrading odorants and pheromones rapidly [123]. So far, there are many ODEs identified in the insect olfactory system, including cytochrome P450s, GSTs, CCEs, UGTs, aldehyde oxidases, epoxide hydrolases, and alcohol dehydrogenase [15,124,125,126]. The first odorant-degrading enzyme (ODE) discovered in insects was a CCE located in the pheromone sensitive sensilla in the antenna of a male silk moth, *A. polyphemus*, in 1981 [21]. In addition to the first ODE, two more CCEs were also found to metabolize pheromones; one located in the wing scales [24], and the other located in integument tissues of both *A. polyphemus* sexes [21]. However, to be considered an ODE, an enzyme must degrade odors and reside in the sensillar lymph. 

CCEs represent the first ODEs discovered to degrade insect pheromones and plant odors and remain the most promising ODE candidates. CCEs that are involved in odorant degrading mainly belong to β-esterases, integument esterases, or mitochondrial and cytosolic CCEs. In *Spodoptera littoralis* (Egyptian cotton leafworm), SICXE10 and SICXE7, two CCEs predominantly expressed in olfactory sensilla, both hydrolyze the female-produced sex pheromones (Z9E11-14:Ac and Z9E12-14:Ac) and the green leaf volatile ((Z)-3-hexenyl acetate) in vitro. SICXE10 and SICXE7 were shown to be expressed in pheromone-sensitive sensilla and short sensilla, and both esterases hydrolyzed Z9E11-14:Ac, Z9E12-14:Ac, and (Z)-3-hexenyl acetate, suggesting that these two CCEs play a role in degrading pheromone and plant odorants [127,128]. Similar studies were conducted on *S. exigua* (Beet armyworm) for SexiCXE11 and SexiCXE14, both showing hydrolysis activity towards host plant volatiles and pheromone esters [31,129].

Two ODEs, Est-6 and JHE-dup, were uncovered in *D. melanogaster* with high expression in the third antennal segment. Est-6 could hydrolyze multiple volatile food esters but had little to no hydrolysis activity towards the pheromone cis-vaccenyl acetate [73,130]. However, JHE-dup, a duplication of the juvenile hormone esterase that had gained new function, showed a 1000-fold higher expression level in the antenna than other tissues and had also shown hydrolysis activity toward multiple food esters [125,131]. In the Japanese beetle, *P. japonica*, one ODE *Pjap*PDE was found to specifically metabolize sex pheromone, R-japonilure. It was also able to hydrolyze the enantiomer (S-japonilure), but at a much slower rate. This enantiomer S-japonilure happened to be an important sex pheromone of a competitive species and a behavioral antagonist for the Japanese beetle [29]. Recently, two paralogous antennae enriched CCEs, *Pxyl*CCE16a and *Pxyl*CCE16c, were identified and characterized in diamondback moth *P. xylostella* [132]. Both CCEs were found to hydrolyze sex pheromone components (Z9-14:Ac and Z11-16:Ac) and plant odorants. Most recently, in the oriental fruit moth *Grapholita molesta*, two β-esterases (GmolCXE14 and GmolCXE21) able to hydrolyze ripe fruit esters were discovered and functionally characterized. In addition, another β-esterase and one integument esterase, GmolCXE1 and GmolCXE5, were found to be able to hydrolyze the sex pheromone (Z/E)-8-dodecenyl acetate [15]. 

## 4. Conclusions

CCEs constitute a multigene family of enzymes that catalyze the hydrolysis of structurally diverse xenobiotics, including pesticides, insect and plant odors, insect pheromones and hormones, as well as environmental pollutants. Studies have suggested that insect CCEs play multiple vital roles in facilitating the adaptation of insects to their chemical environment. Understanding the mechanisms of insect CCE-mediated chemical adaptation will help in advancing the management of insect pests. With recent advances in genome sequencing, X-ray crystallography and functional genomics, our knowledge of insect CCE classification, protein structure characteristics and the dynamic roles of insect CCEs in chemical adaptation will improve by leaps and bounds.

## Data Availability

Not applicable.
